# An investigation of the incidence of post-traumatic stress disorder, turnover intention and psychological resilience among medical staff in a public hospital in China during the outbreak of the omicron variant in the COVID-19 pandemic in 2022

**DOI:** 10.3389/fpsyt.2022.999870

**Published:** 2022-09-06

**Authors:** Cui Jing, Zhang Feng-Hong, Wang Yi-Yan

**Affiliations:** ^1^Stomatology Hospital, School of Stomatology, Zhejiang University School of Medicine, Hangzhou, China; ^2^The Second People’s Hospital of Gansu Province, Lanzhou, China; ^3^West China Hospital of Sichuan University, Chengdu, China

**Keywords:** COVID-19, incidence, post-traumatic stress disorder, turnover intention, psychological resilience

## Abstract

**Objective:**

To investigate the incidence of post-traumatic stress disorder (PTSD), turnover intention and psychological resilience of medical staff during the Outbreak of the Omicron Variant in the COVID-19 pandemic in 2022 and to provide a basis for adopting relevant psychological interventions to reduce medical staff turnover.

**Methods:**

Using the PTSD Checklist-Civilian Version (PCL-C) and a total score ranging from 17 to 85 points, a total score ≥ 38 indicates significant PTSD symptoms and a diagnosis of PTSD. The Chinese version of the Turnover Intention Scale (TIS) has a total score of 6 to 24 points; the higher the score, the stronger the turnover intention. The Chinese version of the Connor-Davidson Resilience Scale (CD-RISC) has a total score of 0 to 100 points, with higher scores indicating a better level of psychological resilience. A total of 443 front-line medical staff working in Chinese public hospitals and still treating all patients normally during COVID-19 were invited *via* the internet to complete a survey from 15 May to 30 May 2022 in China.

**Results:**

The incidence of PTSD was 14.4%, the total turnover intention score was 13.38 ± 4.08, and the total psychological resilience score was 87.16 ± 18.42. The prevalence of PTSD was higher among medical staff who were married, had children, and were worried about being infected; in addition, the PTSD group had a higher level of education, higher turnover intention, and lower psychological resilience than the non-PTSD group. The total scores for turnover intention and fear of being infected were risk factors for PTSD, while a high total psychological resilience score and high education level were protective factors for PTSD; the differences were statistically significant (all *P* < 0.05).

**Conclusion:**

Post-traumatic stress disorder among Chinese medical personnel was associated with the marital status, childbirth, education level, turnover intention, and psychological resilience. Among these factors, psychological resilience might be exploited as a protective factor.

## Introduction

Post-traumatic stress disorder (PTSD) is conceptualized as the “overconsolidation of memories” of prolonged or repeated traumatic events and is defined as a debilitating memory disorder, especially during an outbreak of illness or disaster (1). Its development can affect neuroendocrine disorders and psychiatric symptoms such as immune deficiency, absenteeism, insomnia and nightmares, depression, and even suicide (2, 3). The detection and treatment of PTSD in people after a disaster has become a major concern in medical psychology (4). During the SARS epidemic, medical staff were more susceptible to stress disorders than executives due to the need to cope with the potential risk of infection to themselves and their families (5) and the threat of a shortage of personal protective equipment (6), and some medical staff were afraid to go home after work or even considered quitting (7). COVID-19 has been spreading worldwide over the past 2 years, and the global impact is now growing rapidly with the discovery of the SARS-CoV-2 Omicron variant (B.1.1.529) (8). The Omicron variant has a mutation rate that exceeds other variants by approximately 5–11 times, and preliminary studies suggest that this variant causes an increased risk of human reinfection with the virus compared to other strains of concern (9). Fear of COVID-19 is associated with job dissatisfaction and an increased propensity to leave among healthcare workers (10). The high turnover rate of healthcare workers might have disastrous consequences for international efforts to contain the COVID-19 pandemic, but the possible role of PTSD in increasing the propensity to leave has not been examined.

Psychological resilience is the ability to mobilize one’s own or surrounding protective resources when faced with difficulty or adversity and thus to recover quickly and achieve good adaptation, protecting against negative mental health during challenging times (11). Participation in psychological resilience workshops potentially promotes engagement in positive health behaviors and reduces the incidence of mental health symptoms, especially when implemented prior to repeated trauma exposure (12). In this critical situation, this questionnaire study was performed to understand the incidence of PTSD, turnover intention and psychological resilience among medical personnel in China in the context of the Omicron epidemic, to develop appropriate psychological interventions for the mental health of frontline medical personnel fighting the epidemic and to provide a basis for reducing medical personnel attrition.

## Materials and methods

### Participants

The study was conducted anonymously at the Second People’s Hospital of Gansu Province from 15 May to 30 May 2022 and was approved by the Ethics Committee of the Second People’s Hospital of Gansu Province. The approval number was GSSEY2022-KY014-01. An online questionnaire, including a demographic profile questionnaire, the PTSD Checklist-Civilian Version (PCL-C), the Connor-Davidson Resilience Scale (CD-RISC), and Turnover Intention Scale (TIS), was distributed *via* WeChat, one of the most commonly used chat tools in China. Inclusion criteria were an age>18 years, understanding and consent to participate in the study, voluntary signing the informed consent form, ability to answer the questionnaire using WeChat, and employed as a staff member in a formally designated position who are working in Chinese public hospitals and still treating all patients normally during COVID-19.

### Measurement

#### Demographic information questionnaire

This portion of the question assessed the participants’ gender, age, marital status, fertility status, education level, job title, nature of work, annual household income, experience in the treatment or care of COVID-19, and fear of being infected.

#### Post-traumatic stress disorder Checklist-Civilian Version

The PCL-C, a 17-item self-report PTSD scale, is an internationally accepted screening questionnaire for PTSD. It is divided into 3 dimensions, namely, trauma re-experiencing (5 items), numbness and avoidance (7 items), and increased alertness (5 items). The responses are scored on a 5-point Likert scale, with each entry being scored from 1 to 5 points ranging from (1 = no to 5 = very severe), and the total score ranges from 17 to 85. The higher the score, the more likely PTSD is to occur; a total score of ≥38 indicates significant PTSD symptoms and a diagnosis of PTSD. The Cronbach α for the scale in this study was 0.957.

#### Turnover Intention Scale

The Turnover Intention Scale (TIS) was developed by Michael and Spector (13) in 1982 and was subsequently translated and revised by Li and Li (14), with a Cronbach’s alpha of 0.773 and content validity of 0.677. The TIS main content scale contains three dimensions and six items: the possibility of quitting the current job, finding another job, and getting a different job. Items 1 and 6 constitute the intention to leave, indicating the possibility of quitting the current job; items 2 and 3 constitute the intention to leave I, indicating the motivation to find another job; and items 4 and 5 constitute the intention to leave II, indicating the possibility of obtaining a different job. A 4-point Likert scale is used, with each entry scored on a reverse scale of 1 to 4 points (4 = often to 1 = never), with a total score of 6 to 24 points; the higher the score, the stronger the turnover intention. The Cronbach α for the scale in this study was 0.848.

#### The Chinese version of the Connor-Davidson Resilience Scale

The Chinese version of the CD-RISC, which was developed by Connor-Davidson et al. (15) and contains 25 items in 5 dimensions, was used. The Chinese version was translated and revised in 2007 by Yu et al. (16); this version also has 25 items, but the original 5 dimensions are modified into 3 dimensions, namely, optimism (4 items), self-improvement (8 items), and resilience (13 items). A 5-point Likert scale is used, with each entry scored from 0 to 4 points (0 = never to 4 = always), and a total score of 0 to 100 points, with higher scores indicating a better level of psychological resilience. The Cronbach α for the scale in this study was 0.964.

### Statistical methods

Statistical analyses were conducted using SPSS 25.0 software. Count data are reported as rates (%), and measurement data met the normality test and are presented as M ± SD. Two independent sample *t*-tests and 2 tests were used for comparisons between two groups, and factors with *P* < 0.05 were entered into binary logistic regression model for the multifactor analysis.

## Results

### Demographic information

The demographic information of all subjects is shown in Table 1. A total of 443 front-line medical staff participated in this study, and 64 (14.4%) had PTSD.

**TABLE 1:** General information on the study population (*n* = 443).

Projects	Number of people (n)	Composition ratio (%)
**PTSD**		
Yes	64	14.4
No	379	85.6
**Gender**		
Male	62	14.0
Female	381	86.0
**Age (years)**		
≤35	335	75.6
>35	108	24.4
**Marital status**		
Unmarried	123	27.8
Married	320	72.2
**Fertility status**		
No children	165	37.2
Children	278	62.8
**Education level**		
Specialized	135	30.5
Bachelor’s degree and above	308	69.5
**Title**		
Junior	284	64.1
Senior Intermediate	159	35.9
Nature of work		
Employed by the hospital	138	31.2
Employed by an agency	305	68.8
**Any experience of COVID-19 treatment or care**		
Yes	158	35.7
No	285	64.3
**Fear of being infected**		
Yes	253	57.1
No	190	42.9

### Single-factor analysis of post-traumatic stress disorder

Table 2 shows that the total turnover intention score was 15.69 ± 3.84 for the PTSD group and 13.99 ± 4.00 for the non-PTSD group, and the total psychological resilience score was 79.33 ± 14.07 for the PTSD group and 88.49 ± 18.75 in the non-PTSD group among the 443 medical staff. Compared to the non-PTSD group, the PTSD group had a higher turnover intention and lower psychological resilience (both *P* < 0.05). In addition, the PTSD prevalence was higher among female, married, and childbearing medical staff, and the PTSD prevalence was also higher among medical staff who were highly educated and worried about being infected (all *P* < 0.05).

**TABLE 2:** Correlation analysis of psychological resilience scores with insomnia severity and general information (M ± SD, *n* = 443).

Projects	PTSD		c2/*t* value	*P*-value
	Yes, *n* = 64 (%)	No, *n* = 379 (%)		
Turnover intention (M ± SD)	15.69 ± 3.84	13.99 ± 4.00	5.015	<0.001
Mental toughness (M ± SD)	79.33 ± 14.07	88.49 ± 18.75	−3.732	<0.001
**Gender**				
Male	5 (8.1)	57 (91.9)	2.376	0.123
Female	59 (15.5)	322 (84.5)		
**Age (years)**				
≤35	43 (12.8)	292 (87.2)	2.886	0.089
>35	21 (19.4)	87 (80.6)		
**Marital status**				
Unmarried	10 (8.1)	113 (91.9)	5.497	0.019
Married	54 (16.9)	266 (83.1)		
**Fertility status**				
No children	14 (8.5)	154 (91.5)	7.938	0.005
Children	50 (18.0)	228 (82.0)		
**Education level**				
Specialized	8 (5.9)	127 (94.1)	11.407	0.001
Bachelor’s degree and above	56 (18.2)	252 (81.8)		
**Title**				
Junior	36 (12.7)	248 (87.3)	2.008	0.157
Senior Intermediate	28 (17.6)	131 (82.4)		
Nature of work				
Employed by the hospital	24 (17.4)	114 (82.6)	1.406	0.236
Employed by an agency	40 (13.1)	265 (86.9)		
**Any experience of COVID-19 treatment or care**				
Yes	23 (14.6)	135 (85.4)	0.002	0.961
No	41 (14.4)	244 (85.6)		
**Fear of being infected**				
Yes	47 (18.6)	206 (81.4)	8.141	0.004
No	17 (8.9)	173 (91.1)		

### Multifactor analysis of post-traumatic stress disorder

Using the PTSD status as the dependent variable and a significant factor in the univariate analysis as the independent variable, a binary logistic regression analysis was conducted, and the dichotomous variables were assigned the values shown in Table 3: PTSD status: no = 0, yes = 1; marital status: unmarried = 0, married = 1; fertility status: no children = 0, children = 1; education level: specialist = 0, undergraduate and above = 1. As shown in Table 4, the binary logistic regression results indicated that the total turnover intention and fear of being infected were risk factors for PTSD, and high total psychological resilience score and a high education level were protective factors for PTSD, all with statistically significant differences (all *P* < 0.05).

**TABLE 3:** Table of independent variable assignments.

Variable	Variable name	Assignment
Y	Presence of PTSD	No = 0; Yes = 1
X1	Marital status	Unmarried = 0; Married = 1
X2	Fertility status	No children = 0; Children = 1
X3	Education level	Specialized = 0; Bachelor and above = 1
X4	Fear of being infected	No = 0; Yes = 1

**TABLE 4:** Multifactorial analysis of post-traumatic stress disorder (*n* = 443).

Independent variables	*B*	*SE*	*Beta*	*P-value*	*OR*	*95% CI*
Willingness to leave	0.125	0.040	9.912	0.002	1.134	1.048–1.226
Mental toughness	−0.022	0.009	6.594	0.010	0.978	0.962–0.995
Education level	−0.977	0.425	5.287	0.021	0.376	0.164–0.866
Fear of being infected	0.691	0.317	4.751	0.029	1.996	1.072–3.716

## Discussion

In the present study, the prevalence of PTSD among health care workers was 14.4%, which was higher than the prevalence of PTSD among the general population in China (9.28%) (17). This difference may be because the general public is not directly fighting on the front line of the epidemic and perhaps is not fully aware of the dangers of the new variant of COVID-19 due to a lack of medical knowledge. The results were supported by the findings reported by Petrie et al. (18) and Salehi et al. (19). Although COVID-19 has been endemic worldwide for 2 years, the incidence of PTSD among Chinese medical staff in this study is still high. A probable explanation for this finding is that although many countries have protected against the virus, including through vaccination; the lethality of COVID-19 has been reduced. However, the direction of virus development is difficult to predict because many variables and uncontrollable aspects exist, and the effectiveness of available diagnostic methods, vaccines, and treatments has been reduced (20). The Chinese government has taken a strict and unrelenting approach to the epidemic and has implemented a series of measures to prevent the epidemic for the whole population (e.g., implementing regular nucleic acid testing and criminal liability for obstruction of epidemic prevention) (21). Therefore, as long as this epidemic is present, all people should be aware of it. On the other hand, PTSD is not immediately apparent in medical personnel during disasters. A study of 7,393 health care workers showed that between 2 and 19% reported symptoms of PTSD within 1–3 years after the outbreak (22), supporting the conclusion that PTSD is not always immediately apparent. Therefore, the increasing number of confirmed and suspected cases, heavy workload, depletion of personal protective equipment, and extensive media coverage may have all contributed to these health care workers’ mental burden, although COVID-19 has been a global pandemic for 2 years.

The prevalence of PTSD in women in this study was twice that of men, consistent with a large number of studies that have shown a higher prevalence of PTSD in women (23, 24). Among the sex –related factors, sex hormone levels, such as testosterone, estradiol, and progesterone, are postulated to be associated with the development of PTSD, and these factors may directly influence the risk of PTSD through epigenetic mechanisms, putting women at a particularly high risk of developing PTSD (25). On the one hand, although the proportion of women in this study was greater than the proportion of men, the China Health and Health Statistical Yearbook (2021) reported that 72.4% of health technicians nationwide were women at the end of 2020 (26). Furthermore, according to the National Health and Wellness Commission of China, 67% of the 42,600 medical personnel assisting in Wuhan at the time of the COVID-19 outbreak were female medical personnel (27) showing that women are gradually becoming the mainstay of China’s medical profession. Therefore, the gender distribution of the subjects in this study is consistent with the current gender distribution of Chinese medical personnel and may reflect the incidence of PTSD and psychological resilience of Chinese medical personnel in the face of epidemics to a certain extent. On the other hand, in a developing country, such as China, socioeconomic factors and gender roles must be considered to understand this association. In China, traditional thinking holds that men earn money outside the home while women take care of the family, but as society changes and women become more involved in social work, women tend to experience more work and family conflicts than men. Women are more likely to feel the tension between their careers and the demands of their families. Because they often have more responsibility for their families, children, and patients, this dilemma of trying to achieve an ideal work-life balance may make women feel like failures, potentially increasing their vulnerability to PTSD. In China, therefore, female medical professionals are not only biologically and genetically influenced but are also at higher risk of PTSD due to their specific cultural environment and gender roles.

The higher incidence of PTSD in married medical personnel is not entirely consistent with a meta-study (28) that reported a higher incidence of psychological stress in unmarried medical personnel than in married personnel during the COVID-19 epidemic, possibly due to cultural constraints on the incidence of PTSD that vary between countries (29–31). A study by Li et al. (32) assessing Chinese medical team members during the COVID-19 epidemic supports our hypothesis.

Similar to the results from the study by Yasmin et al. (33), the present study concluded that the incidence of PTSD was higher among medical personnel with children. Numerous studies have reported increased mental health risks associated with the presence of children in the family during COVID-19 (34–36), and concerns about children’s health potentially contribute to higher rates of PTSD among medical staff in families with children because children have relatively less immunity than adults.

A higher education level and fear of infection were risk factors for PTSD in health workers (*P* < 0.05) Giorgi et al. (37) reported that PTSD was more likely to affect health workers during COVID-19, especially frontline workers with higher educational backgrounds. This result may be due to the increased speed of transmission and infectiousness of the virus in the face of ongoing mutations of the new coronavirus and the fact that more educated health workers are more likely to have access to information about COVID-19 and to feel more afraid of the virus. Fear of disease is a risk factor for psychological stress during a pandemic (38), consistent with the findings of the present study. In contrast, less educated individuals may not be aware of the potential hazards of a pandemic and therefore may exhibit a lower PTSD incidence (39).

In addition, the PTSD group had a higher total score for turnover intention and a lower total score for psychological resilience than the non-PTSD group. The differences between COVID-19 illness and turnover intention and psychological resilience were significant (both *P* < 0.05). In this case, turnover intention was a risk factor, while psychological resilience was a protective factor. Previous studies of the SARS outbreak have shown that health care workers often experienced isolation after being involved in treating infected patients, and studies of Chinese hospital staff reported higher levels of stress among isolated health care workers who expressed reluctance to work or considered quitting (7). Since the outbreak, different occupations have been hit differently, with health care workers facing a greater occupational risk than others, increasing their likelihood of infection. In the COVID-19 context, we used the O*Net (40) definition of occupational risk that is divided into two components: (i) the level of physical contact with other individuals and (ii) the frequency of exposure to possible diseases or infections. Several researchers have found that healthcare workers are among those at the highest risk of infection (41, 42). In addition to extrinsic environmental factors, researchers have found that individual factors such as workability and perceived threat to work potentially influence employees’ decisions to continue working (43, 44). The results of a Korean study showed that in a pandemic context, where viruses are constantly mutating, healthcare workers still face uncertainty in terms of competence and risk, as well as threats to their safety, generating burnout and a lower willingness to retain their jobs (41). In addition, the study showed that a decrease in career retention intentions implies a shrinking healthcare workforce, which would be a major obstacle to overcoming COVID-19.

The findings of the present study on psychological resilience as a protective factor for PTSD are consistent with the findings reported by Liu et al. (45) for Chinese medical personnel during COVID-19. Resilient individuals tend to be optimistic and adaptive, with high resilience positively correlated with well-being and negatively correlated with perceived stress (46); resilient individuals are able to maintain perspective and daily functioning in the face of problems, representing the strength to overcome obstacles with competence and hope (47). Some studies have shown that resilience predicts secondary traumatic stress in medical personnel and that psychological resilience is protective against traumatic stress (48), while psychological resilience also mediated the relationship between COVID-19 stress experiences and acute stress disorder in a study of 7,800 university students (49). Furthermore, studies on psychological resilience have reported a protective effect on turnover intentions, with psychological resilience reducing burnout and turnover rates (50). We should identify risk and protective factors that are important to reduce the occurrence of PTSD. A review of guidelines and recommendations issued during the COVID-19 pandemic by Halms et al. (51) also showed that structural social support and improvements in the work environment were important for health workers in the fight against the epidemic. We therefore also recommend that the mental health of medical staff be carefully monitored and that health care organizations provide support to medical staff with sufficient flexibility to prevent health care system breakdown in response to a pandemic.

However, several limitations of this study must be acknowledged. First, we used a snowball sampling method to recruit medical staff online, which may have resulted in a sampling bias, as some older medical staff do not use social networks; this limitation was evident in our sample, as a larger proportion of participants were under 35 years of age. This recruitment method may also have resulted in a skewed gender demographic distribution, with a larger proportion of women in the current sample and little data available from men, which also limits the generalizability of the findings. Second, as this study recruited subjects working in only one public hospital, some bias in the proportion of medical staff with or without experience in COVID-19 treatment or care was also present, and the findings should be validated in future studies in multiple centers. Furthermore, the cross-sectional design of the current survey did not allow for a causal relationship to be established, and the short duration of the survey did not allow for the effective validation of whether a dynamic change in PTSD prevalence occurs with the development of COVID-19.

## Conclusion

In summary, this study revealed a high prevalence of PTSD among health care workers during COVID-19. Emphasis on the screening and treatment of PTSD is important for maintaining the physical and mental health of healthcare workers during the epidemic and to reduce staff turnover. The findings also revealed that being married with children and a fear of being infected were associated with a higher prevalence of PTSD among healthcare workers and that the PTSD group had a higher level of education and turnover intention in the workforce and lower psychological resilience than the non-PTSD group. Associated risk factors included a high turnover intention in the profession and fear of infection. In addition, tolerance of psychological resilience and a high level of literacy were protective factors for PTSD. Focusing on gender differences, culture and other aspects of the lives of staff enable a better understanding and perception of their psychological experiences (52). Hospital administrators should actively improve the psychological resilience of volunteers, cultivate optimism and resilience, and use psychological resilience as a positive psychological resource to play an active role in reducing the incidence of PTSD and turnover intention among front-line medical staff, which is important for responding to and providing relief during major disasters. In addition, government disaster preparedness plans should include provisions and interventions to address mental health issues among medical staff.

## Data availability statement

The original contributions presented in this study are included in the article/supplementary material, further inquiries can be directed to the corresponding author.

## Ethics statement

The studies involving human participants were reviewed and approved by the Medical Ethics Committee, and Second People’s Hospital of Gansu Province. The patients/participants provided their written informed consent to participate in this study.

## Author contributions

CJ and ZF-H: conceptualization, methodology, investigation, data collection, statistical analysis, manuscript preparation, writing – review and editing, supervision, and revision. WY-Y: conceptualization, methodology, investigation, data collection, and manuscript preparation. All authors contributed to the article and approved the submitted version.

## Abbreviations

PTSD: post-traumatic stress disorder
PCL-C: PTSD Checklist-Civilian Version
TIS: Turnover Intention Scale
CD-RISC: Connor-Davidson Resilience Scale.
